# Change in eating habits during the Spanish COVID-19 pandemic lockdown: evidence for a sample of university community

**DOI:** 10.7717/peerj.14244

**Published:** 2023-01-25

**Authors:** David Cantarero Prieto, Paloma Lanza-León, Patricia Moreno, Carla Blázquez-Fernández, Javier Lera, Francisco-Jose Gonzalez-Diego, Irene González Rodríguez

**Affiliations:** 1 Universidad de Cantabria, Santander, Spain; 2 Valdecilla Biomedical Institute Research (IDIVAL), Santander, Spain; 3 Universidad de La Rioja, La Rioja, Spain; 4 Cantabria Health Service, Santander, Spain

**Keywords:** Eating habits, COVID-19, University students

## Abstract

**Background:**

The stress and anxiety caused by COVID-19 lockdown may have changed the eating habits of the population. Our aim is to assess the eating changes that have taken place due to the pandemic.

**Methods:**

Data were collected through an electronic survey created by the Health Economics Research Group of the University of Cantabria and IDIVAL and conducted between 14/01/2021 and 19/02/2021. A total of 1,417 responses were recorded, but only 507 complete observations were considered. We carried out a cross-sectional analysis through ordered probit regressions.

**Results:**

The improvement in post-confinement eating habits is associated with higher income level, better self-assessed health status and more physical activity. The worsening of eating habits is associated with having a certain level of nomophobia or the fear of contagion.

**Conclusions:**

Our analysis can be used for designing and implementing new strategies to overcome the negative spill overs of the COVID-19 pandemic and improve the dietary patterns.

## Introduction

On 12 March 2020, the World Health Organization (WHO) declared the coronavirus outbreak a global pandemic (Organización Panamericana de la Salud, 2020). Without vaccines to prevent infection and no effective treatment for the infected, most of the countries decided to implement different levels of lockdown. For example, access to supermarkets, shops, and leisure-time places were restricted. These restrictions varied from country to country and generally depended on the COVID-19 incidence level. In Spain, a state of alarm was declared to implement a though and generalized lockdown. Nobody was permitted to leave home except for buying essential goods or those people engaged in essential activities. It came into force on 14 March at 12 midnight (Gobierno de España, 2020a). In general, it was only possible to go out briefly to buy food, hygiene, cleaning, or other essentials goods, to take out the rubbish and to walk the dog, if necessary. The rest of the time, staying at home was mandatory. After 3 months, on 21 June, Spain exited the state of alarm, and the President declared the beginning of the “new normality” (Gobierno de España, 2020b) following the end of the first wave of the pandemic. Nevertheless, in the “new normality” Spanish regions continued suffering from the pandemic and implemented different restrictions depending on the COVID-19 incidence level. All the restrictions aimed to limit interpersonal contact in order to reduce the risk of transmission.

Many experts have expressed their concern about the long-term effects that lockdown may have on physical and mental health (Cheval et al., 2021; Ausín et al., 2021) due to changes in peoples’ behaviors. Some of them that might negatively affect our health include an increase in unhealthy eating, excessive sleeping, and a decrease in physical activity (Knutson, 2012; Knell et al., 2020; Censi et al., 2022).

Dietary habits became worse during the pandemic; people consumed more ultra-processed food whose nutritional quality is lower, more snacks between meals, more alcohol, or less fresh food (Jacob et al., 2021; Deschasaux-Tanguy et al., 2021; Ammar et al., 2020; Villaseñor Lopez et al., 2021; Al-Domi et al., 2021; Tan, He & MacGregor, 2020). These new habits do not match with the WHO characterization of health eating and the recommendations for a healthy diet during the COVID-19 pandemic (Muscogiuri et al., 2020). The WHO definition of healthy dietary habits a diet includes a high level of fruits, vegetables, fresh food and low consumption levels of fats, oil, salt and sugar (World Health Organization (WHO), 2020). Recent studies show that those diets which main components are fruits and vegetables are consistently associated with a range of health benefits such as lower risk of diabetes, stroke and other coronary diseases (Dauchet et al., 2006; Wang et al., 2014). To sum up, unhealthy eating is understood as the increased consumption of sweets, soft drinks alcohol and fast food. On the other hand, an increase in vegetables, legumes and fruit results in an improved diet. Moreover, problems can be linked to each other: reduced physical activity can increase consumption of unhealthy foods “out of boredom” (Catucci, Scognamiglio & Rossi, 2021). The lockdown restricted the number of hours allowed for outdoor physical activity, while gyms were closed, which has led to a decrease in the physical activity level (Joseph et al., 2021).

The 2020 WHO guidelines on physical activity and others organism highlight the importance of regularly undertaking aerobic and muscle strengthening activities (Centers for Disease Control and Prevention (CDC), 2022; World Health Organization (WHO), 2010; Bull et al., 2020).

Some studies point out that people report important barriers for weight management (Robinson et al., 2021; Warburton & Bredin, 2017; Yu, Malik & Hu, 2018). Nevertheless, this effect depended on age (McCarthy, Potts & Fisher, 2021). In addition, these unhealthy changes may promote the risk of chronic non-communicable diseases (Severi & Medina, 2020).

Physical activity is known to have beneficial effects on health (Warburton & Bredin, 2017; Yu, Malik & Hu, 2018). Access to fresh food was also restricted at an early stage which, with the increased stress resulting from the pandemic, can have a major impact on individual habits (Mattioli et al., 2020). Evidence (El Ansari, Adetunji & Oskrochi, 2014) shows that consuming “unhealthy” food (*e.g*., sweets, cookies, snacks, fast food, *etc*.) is significantly associated with depressive symptoms for women and men and perceived stress in the case of women. Thus, consuming “healthy” food would be associated with lower depressive symptoms and lower perceived stress among female and male students. Stress and anxiety have been shown to increase intake of alcohol and sugary foods, and energy imbalance is also likely due to decreased energy expenditure during lockdown (Mattioli et al., 2020). It has also been found that boredom can predict changes in eating behaviors independently of other negative emotions (Moynihan et al., 2015). However, some healthy habits may have been acquired during the pandemic, such as spending more time cooking or reducing fast food consumption (Galafate, 2020).

Another habit change is the time of mobile phone use or “screen time” Some authors (Tayhan Kartal & Yabancı Ayhan, 2021; Frieiro, González-Rodríguez & Domínguez-Alonso, 2022), suggest that students’ phone and internet addiction significantly affect eating disorders and overweight. Excessive use of these technologies can also affect mental health. Specifically, those most affected by this problem are adolescents (Park et al., 2022). Some papers suggest that the evidence is not consistent enough to prove a true relationship (Griffiths et al., 2010) while others find a positive relationship (Cao et al., 2011; de Wit et al., 2011; Khouja et al., 2019; Hinojo-Lucena et al., 2019). Most of the studies has a cross sectional design and methodology is not strong enough (Teychenne, Costigan & Parker, 2015). A study by Moitra & Madan (2022) focused on the impact of screen time on the eating habits, physical activity, sleeping habits and depressive symptoms during the COVID-19 pandemic. They showed that half of the interviewed teenagers had poor sleeping habits. Moreover, the authors suggest that screen time was associated with a higher consumption of snacks and poorer eating habits and lower levels of physical activity. A similar review by Hale & Guan (2015) found that screen time is negatively associated with sleep outcomes. The results varied depending on the type of screen exposure, age, gender and the day of the week. Nevertheless, this study had some limitations: causal association was not confirmed, data did not contain enough information regarding the use of simultaneous use of multiple screens, and the measure of screen time did not take into consideration the reasons behind that time or the use.

After lockdown and the various waves, changes in eating consumption habits are likely to continue, which are key aspects for the development of strategies and actions to promote a correct diet in the context of a healthy lifestyle (America Heart Association, 2022). Our aim is to assess the eating changes that have taken place because of the pandemic. We hypothesize that the COVID-19 lockdown had some side effects such a decrease in the physical activity levels, the loss of habits and routines, the increase of stressful situations, social isolation and boredom, worsened social networks or increased mobile phone use. These side effects vary depending on several factors: biological, psychological and sociocultural. All in all, behaviors might have changed due to the pandemic.

The article is organized as follows: “Materials and Methods” describes the data used and presents the methodology, “Results” shows the main results of the analysis, “Discussion” discusses these results and “Conclusions” presents the study’s findings.

## Materials and Methods

Three important issues in this section are the data, the empirical strategy and the variables employed in the paper. This information is divided into the following subtitles: “Participants”, “Procedure”, “Measurements and statistical analyses”.

### Participants

We explore the change in eating habits before and during the COVID-19 pandemic, here measured by dietary changes, using a cross-sectional analysis through ordered probit regressions. Precisely, students and workers from a Spanish public University, the University of Cantabria, constitute our sample.

### Procedure

Data was collected through an electronic survey that we created using LimeSurvey software (LimeSurvey, 2022). The survey was conducted in the period from 14/12/2020 to 19/02/2021, in which Cantabria was at a health alert level 4 and in the midst of the third wave (Gobierno de Cantabria, 2021). It is important to be highlighted that this survey received the approval by the University of Cantabria Academic Committee (November 26, 2020) (Ethical Application Ref: *C. Proyectos 16/2020*). All in all, the questionnaire used in this study was completely anonymous. In addition, since the survey is conducted online, the respondent information sheet appears on the survey presentation screen. After that first screen, the informed consent form is included. Hence, the informed consent of the participants has been obtained by clicking the “Start survey” button.

### Measurements and statistical analyses

In this article, we are dealing with an ordered outcome dependent variable, so the adequate method has to allow managing this characteristic. Hence, ordered probit regression is used due to these models are considered in the case of studies with more than two outcomes of an ordinal dependent variable that are ordered categories. In this study, as specified below, the ordinal dependent variable has three possible outcomes; change the eating habits to worse before and during the COVID-19 pandemic (alternative 0), maintain more or less the same eating habits (alternative 1) or to improve their eating habits (alternative 2).

At is well known the interpretation of the results in this kind of models require more than just examining the direction and level of statistical significance for the coefficient estimates and it is interesting to obtain the magnitude of these effects.

The most common way to interpret the results of an ordered probit model is to compute predicted probabilities based on the results of the analysis. Because the predicted probability of falling in any of the categories of the dependent variable is a nonlinear function of the independent variables, computing predicted probabilities requires setting every independent variable at some value. It could be thought of as creating a descriptive profile for a case in the dataset and computing a predicted probability for someone with that profile falling into each activity level. One can evaluate how different independent variables impact changes in predicted probabilities by changing features of the profile and recomputing those probabilities.

Let us to define an index model for a single latent variable ${y^*}$, unobservable for the investigator, the only we can observe is if it crosses thresholds.

(1) $$y_i^* = x_i^T\beta + {u_i}$$

(2) $${y_i} = j\; \; if\; {\alpha _{j - 1}} < y_i^* < \; {\alpha _j}$$

Here, the ${x_i}$ are the explanatory variables considered in the analysis for each individual $i$. These variables are socio-demographic and social-isolation factors defined in Table 1.

**Table 1:** Variables used, coding and main descriptive statistics.

Variable		Coding	Mean	S.D.
*Dependent variable*	*Dietary changes*	0: if the individual reported a change in his/her eating habits before and during the COVID-19 pandemic (worse); 1 if the individual declared to have maintained more or less the same eating habits and 2 if the individual reported to have improved the eating habits		
*Socio-demographic* *factors*	*Female*	1: female; 0: male	0.635	0.482
	*Age*	Aged of the individual (years)	35.012	15.614
	*Student*	1: if the respondent is a student; 0: otherwise	0.550	0.498
	*Single*	1: if the individual is single; 0: otherwise	0.596	0.491
	*Self-assessed health (SAH)*	1: if the respondent assessed his/her general health status as good or very good; 0: otherwise	0.270	0.445
	*Sports habits*	1: if the individual reported an improvement in his/her sports habits before and during the COVID-19 pandemic; 0 otherwise	0.179	0.384
	*Income_1*	1: if the household annual income is less than 7,847 euros; 0 otherwise	0.197	0.139
	*Income_3*	1: if the household annual income is more than 26,747 euros but less than 34,419 euros; 0 otherwise	0.174	0.379
*Social Isolation factors*	*Alone*	1: if the individual lives alone; 0: otherwise	0.095	0.293
	*Mobile dependence*	1: if the respondent can be less than an hour without looking at the mobile phone; 0 otherwise	0.136	0.343
	*Emergencies*	1: if the individual reported that does not leave home expect for emergencies; 0 otherwise	0.101	0.301

**Note:**

Observations = 507. Source: Authors’ elaboration.

Thus, the probability that observation $i$ will select alternative $j$ (worse, the same or better eating habits worse before and during the COVID-19 pandemic) is:

(3) $${P_{ij}} = P\left( {{y_i} = j} \right) = P\left( {{\alpha _{j - 1\; }} < y_i^* < \; {\alpha _j}} \right) = F\left( {{\alpha _j} - x_i^T\beta } \right) - F\left( {{\alpha _{j - 1}} - x_i^T\beta } \right)$$

In this case, we are going to assume that F is the standard normal cdf, so we have the ordered probit specification. This kind of models are usually estimated by maximum likelihood methods, as we do, using the statistic software Stata-14. In order to interpret the results, we have also obtained the marginal effects. Here, the marginal effect of an increase in a regressor ${\boldsymbol{x}_{\boldsymbol{r}}}$ on the probability of selecting alternative j is:

(4) $$\displaystyle{{\partial {P_{ij}}} \over {\partial {x_{ri}}}} = \left\{ {{F}^{\prime}\left( {{\alpha _{j - 1}} - x_i^T\beta } \right) - {F}^{\prime}\left( {{\alpha _j} - x_i^T\beta } \right)} \right\}{\beta _r}$$

## Results

We start this section with the main descriptive statistics of our analytical sample. We obtained a sum of 1,417 answers (registered). However, some of them were not complete and we decided not to include them in the study. Finally, we could work with 507 observations. A total of 63.5% of the sample were women and in average, the respondents have 35 years old. A total of 55% of these people were students and 59.9% were single. A total of 17.9% report an improvement in his/her sports habits before and during the COVID-19 pandemic while 9.5% lived alone. Finally, a total of 13.6% declared to have certain mobile phone dependence and 10.1% reported not to leave home except for emergencies.

Table 1 contains variables used, coding and its main descriptive statistics.

Next, we present the empirical findings obtained from our ordered probit regression (Table 2). We present both the Odds Ratios (OR) and their associated 95% Confidence Interval (CI). Furthermore, it is important to evaluate the probabilities that the independent variables “fall” within each category because they are dichotomous or dummy variables, and intrinsic continuous variables (regression factors). This is achieved with the results of marginal effects in Table 3.

**Table 2:** Results: ordered probit regression.

Variable		Dietary changes		
		Coef.	95% CI	*p*-value
*Socio-demographic* *factors*	*Female*	−0.110	[−0.327 to 0.106]	0.59
	*Age*	0.001	[−0.010 to 0.011]	0.99
	*Student*	−0.175	[−0.439 to 0.088]	0.08
	*Single*	0.003	[−0.320 to 0.325]	0.91
	*Self-assessed health*	0.248	[0.011–0.484]	0.01
	*Sports habits*	0.764	[0.487–1.041]	0.00
	*Income_1*	−0.380	[−0.807 to 0.046]	0.07
	*Income_3*	0.319	[0.038–0.600]	0.01
*Social isolation factors*	*Alone*	0.160	[−0.223 to 0.543]	0.44
	*Mobile dependence*	−0.290	[−0.592 to 0.012]	0.07
	*Emergencies*	−0.346	[−0.690 to −0.002]	0.01
*Cut1*		−0.972	[−1.569 to −0.374]	
*Cut2*		1.074	[0.474–1.674]	

**Note:**

CI, Confidence Interval; Observations = 507.

**Table 3:** Results: average marginal effects.

Variable			Dietary changes		
			dy/dx	95% CI	*p*-value
*Socio-demographic* *factors*	*Female*	1	0.027	[−0.025 to 0.078]	0.59
		2	−0.001	[−0.006 to 0.004]	0.87
		3	−0.026	[−0.076 to 0.025]	0.59
	*Age*	1	0.000	[−0.003 to 0.002]	0.99
		2	0.000	[0.000–0.000]	0.99
		3	0.000	[−0.002 to 0.003]	0.99
	*Student*	1	0.042	[−0.021 to 0.105]	0.08
		2	−0.002	[−0.009 to 0.006]	0.86
		3	−0.041	[−0.101 to 0.020]	0.08
	*Single*	1	−0.001	[−0.078 to 0.077]	0.91
		2	0.000	[−0.003 to 0.003]	0.92
		3	0.001	[−0.074 to 0.075]	0.91
	*Self-assessed health*	1	−0.059	[−0.116 to −0.003]	0.01
		2	0.002	[−0.009 to 0.013]	0.86
		3	0.057	[0.002–0.112]	0.01
	*Sports habits*	1	−0.183	[−0.252 to −0.115]	0.00
		2	0.007	[−0.026 to 0.040]	0.86
		3	0.177	[0.114–0.239]	0.00
	*Income_1*	1	0.091	[−0.011 to 0.193]	0.07
		2	−0.003	[−0.020 to 0.013]	0.86
		3	−0.088	[−0.190 to 0.011]	0.07
	*Income_3*	1	−0.077	[−0.144 to 0.009]	0.01
		2	0.003	[−0.011 to 0.017]	0.86
		3	0.074	[0.009–0.138]	0.01
*Social isolation factors*	*Alone*	1	−0.038	[−0.130 to 0.054]	0.44
		2	0.001	[−0.006 to 0.009]	0.87
		3	0.037	[−0.052 to 0.126]	0.44
	*Mobile dependence*	1	0.070	[−0.003 to 0.142]	0.07
		2	−0.003	[−0.015 to 0.010]	0.86
		3	−0.067	[−0.137 to 0.003]	0.07
	*Emergencies*	1	0.083	[0.001–0.165]	0.01
		2	−0.003	[−0.018 to 0.012]	0.86
		3	−0.080	[−0.160 to 0.000]	0.01

**Note:**

CI, Confidence Interval; Observations = 507.

We can observe that the female indicator variable produces a coefficient estimate that is not statistically significant, the same occurs with the age, the civil status or if the individual is student or worker or living alone but all of the remaining slope coefficients are statistically significantly different from zero. We have maintained these variables because even not being individually significant, the full model is jointly significant.

It can be observed that the improvement in post-confinement eating habits is associated with higher income level, better health (SAH) and improvements in sport activities. On the other hand, the worsening eating habits is more associated with having a certain dependence on a mobile phone or staying at home and not leaving except for emergencies. Likewise, it can be seen that the intercepts (cut 1 and cut 2) are significant, which indicates that the categories used (in the dependent variable, improvement, maintenance and worsening of eating habits) are adequate and it is not necessary to merge some of them in one.

Reporting good or very good health status additively increases the probability of being in the healthiest eating habits outcome classification by 5.7% points. Having good sports habits also increases the probability of being in the healthiest eating habits outcome classification by 17.7% points while those belonging to the lowest income level reduces the probability of being in the healthiest eating habits outcome classification by 8.8% points. This reduction in the probability is of 6.7% points in case of having mobile phone dependence and 8% points declaring not to leave home except for emergencies.

## Discussion

During lockdown, the two factors that most affect physical and psychological well-being are psychosocial stress and loss of habits and routines (Wang et al., 2020). Those factors may persist for a long time after restrictive measures of lockdown and there may be a gender difference, with greater symptoms of depression, anxiety, and feelings of loneliness in women (Ausín et al., 2021). In our data we have not seen significant gender differences in changes in eating habits, as shown in Table 2, although other authors (Pérez-Rodrigo et al., 2020) have observed differences during lockdown. This may be because the gender differences observed are related to age and in our case the sample is a relatively young population. We also didn’t observe differences in living alone, which may be because in the survey period with a level 4 alarm state some mobility was allowed.

In this study, carried out on a convenience sample during the post-lockdown period of the COVID-19 pandemic in Spain, changes in dietary habits in both directions, both positive and negative changes, as reported by other authors (Instituto de Alimentación (INDA) del Ministerio de Desarrollo Social, 2020; Di Renzo et al., 2020), were observed. Positive changes in dietary habits were significantly associated with higher income levels, better self-perceived health, and improved sporting habits. These healthier eating changes were seen during the months of lockdown with increased consumption of fresh food, vegetables, and dairy products (Ministerio de Agricultura, P. y A, 2020) and increased purchase of fruit and vegetables (Revista ConsUCE, 2020), as well as a decrease in foods considered unhealthy (ABC Los Productos de Mercadona Que Pierden Adeptos Tras El Cambio de Hábitos Por El Confinamiento, 2020). On the other hand, negative changes are related to increased dependence on mobile phone use and staying at home. There is growing evidence of a link between food consumption and psychological health in adults, with anxiety and stress being closely linked to increased consumption of ultra-processed products, sweets, and chocolates (Ramón-Arbués et al., 2019). Sleep patterns in adolescents and young adults have also been shown to be altered during the pandemic (Zhou et al., 2020), and these sleep patterns have been shown to modulate the secretion of several hormones that regulate appetite and increase the secretion of the stress hormone cortisol (Gutierrez & Willoughby, 2010). One of the interesting findings of one study (Wang et al., 2020) was that those with access to the internet and smartphones were young people, and they had higher scores on a scale that measures insomnia, probably because they received more news about the evolution of the disease and the number of deaths that were added every day, which kept them in constant anxiety.

Additionally, there is not a clear consensus in the relationship between “screen time” and health outcomes. Our work suggest how negative changes in eating habits are related to increased mobile phone use. Another study (Delfino et al., 2018) proved that a high use of devices was associated with higher consumption levels of snacks, fried foods, sweets and lower levels of physical activity in adolescents.

We have also observed a relationship between sporting habits and improved diet. King et al. (2012) suggested that an increase of physical activity also increases the energy expenditure with compensatory responses that could lead to higher levels of food intake. They also prove that physiological, psychological and behavioral factors intervene in the relationship between exercise and appetite. In the same way, caloric restrictions may lead to lower compensatory responses in physical activity (Redman et al., 2009).

It is worth remembering that the situation in the first days of lockdown prompted the development of some specific recommendations for dietary care and an encouragement of physical activity at home (Rodríguez, Crespo & Olmedillas, 2020), this activity being key to mitigating the psychological impact of the pandemic (Brooks et al., 2020).

This study has several strengths. The survey data were collected in the middle of the third wave in Cantabria, which provides up-to-date and not outdated information. In addition, the results obtained on the link between mobile phone dependence and eating habits open the door to new lines of research: the impact of excessive use of technologies on health according to their age. Finally, we provide new empirical evidence to support the promotion of good eating and sporting habits.

Nevertheless, we would like to point out the existence of some limitations. The sample chosen, using in part the “snowball method”, with a relatively young population from the university environment, although not exclusively, and with internet dominance, may not represent the Spanish population. The study is a cross-sectional study, so we do not know the duration of the observed changes.

Some future lines of research arise from this study. Firstly, it is necessary to continuously evaluate the implementation of public policy aimed at developing programs of dietary prevention and education, as well as physical activity, specifically targeted and programmed for each age group. Secondly, more evidence is needed to assess the relationship and possible reverse causality in several fields such as dietary habits, use of technology and health outcomes. Thirdly, although the efforts by De Veirman, Cauberghe & Hudders (2017), who tries to disentangle the role of influencers in people perceptions, and the one by Brown et al. (2022) on the mental side effects, more evidence on the effect of internet or influencers on lifestyles and mental health is needed. Thus, more studies are needed in this field to assess the true impact on habits and the health outcomes associated. Finally, long-term effects of the changes in dietary habits, changes physical activity levels and changes in communication among individuals due to the COVID-19 lockdown need to be addressed.

## Conclusions

Overall, the results obtained in this article can be used for designing and implementing new strategies to identify and overcome the negative spill overs of the COVID-19 pandemic specially the dietary patterns. Moreover, this study also calls for a more comprehensive evaluation of adolescents’ symptoms of addiction to technology in order to be able to prevent and intervene correctly and the side effects this addition might have. Furthermore, our findings identify factors associated with improved or worsening eating habits that can be used to formulate interventions to improve the health of general population by prioritizing the most vulnerable groups. We recommend the design, implementation, long-term follow up and continuous evaluation of strategies to facilitate the sustainability of positive changes in terms of diet and to implement measures to reverse negative changes.

## Supplemental Information

10.7717/peerj.14244/supp-1 Supplemental Information 1 Raw data. The survey used to collect the data: the different sections of the survey, including personal information, social distancing, health status and eating habits.

10.7717/peerj.14244/supp-2 Supplemental Information 2 Questionnaire in Spanish.

10.7717/peerj.14244/supp-3 Supplemental Information 3 Questionnaire in English.

## Additional Information and Declarations

### Data Availability

The following information was supplied regarding data availability: The raw data is available as a Supplemental File.

## References

[ref-1] ABC Los Productos de Mercadona Que Pierden Adeptos Tras El Cambio de Hábitos Por El Confinamiento (2020). ABC Comunidad Valenciana. https://www.abc.es/espana/comunidad-valenciana/abci-coronavirus-productos-mercadona-pierden-adeptos-tras-cambio-habitos-confinamiento-202004221959_noticia.html.

[ref-2] Al-Domi H, AL-Dalaeen A, AL-Rosan S, Batarseh N, Nawaiseh H (2021). Healthy nutritional behavior during COVID-19 lockdown: a cross-sectional study. Clinical Nutrition ESPEN.

[ref-28] America Heart Association (2022). Healthy lifestyle. https://www.heart.org/en/healthy-living/healthy-lifestyle.

[ref-3] Ammar A, Brach M, Trabelsi K, Chtourou H, Boukhris O, Masmoudi L, Bouaziz B, Bentlage E, How D, Ahmed M, Müller P, Müller N, Aloui A, Hammouda O, Paineiras-Domingos L, Braakman-Jansen A, Wrede C, Bastoni S, Pernambuco C, Mataruna L, Taheri M, Irandoust K, Khacharem A, Bragazzi N, Chamari K, Glenn J, Bott N, Gargouri F, Chaari L, Batatia H, Ali G, Abdelkarim O, Jarraya M, El Abed K, Souissi N, Van Gemert-Pijnen L, Riemann B, Riemann L, Moalla W, Gómez-Raja J, Epstein M, Sanderman R, Schulz S, Jerg A, Al-Horani R, Mansi T, Jmail M, Barbosa F, Ferreira-Santos F, Šimunič B, Pišot R, Gaggioli A, Bailey S, Steinacker J, Driss T, Hoekelmann A, On Behalf of the ECLB-COVID-19 Consortium (2020). Effects of COVID-19 home confinement on eating behaviour and physical activity: results of the ECLB-COVID-19 international online survey. Nutrients.

[ref-4] Ausín B, González-Sanguino C, Castellanos M, Muñoz M (2021). Gender-related differences in the psychological impact of confinement as a consequence of COVID-19 in spain. Journal of Gender Studies.

[ref-5] Brooks SK, Webster RK, Smith LE, Woodland L, Wessely S, Greenberg N, Rubin GJ (2020). The psychological impact of quarantine and how to reduce it: rapid review of the evidence. Lancet.

[ref-6] Brown O, Smith LGE, Davidson BI, Ellis DA (2022). The problem with the internet: an affordance-based approach for psychological research on networked technologies. Acta Psychologica.

[ref-7] Bull FC, Al-Ansari SS, Biddle S, Borodulin K, Buman MP, Cardon G, Carty C, Chaput J-P, Chastin S, Chou R, Dempsey PC, DiPietro L, Ekelund U, Firth J, Friedenreich CM, Garcia L, Gichu M, Jago R, Katzmarzyk PT, Lambert E, Leitzmann M, Milton K, Ortega FB, Ranasinghe C, Stamatakis E, Tiedemann A, Troiano RP, van der Ploeg HP, Wari V, Willumsen JF (2020). World health organization 2020 guidelines on physical activity and sedentary behaviour. British Journal of Sports Medicine.

[ref-8] Cao H, Qian Q, Weng T, Yuan C, Sun Y, Wang H, Tao F (2011). Screen time, physical activity and mental health among urban adolescents in China. Preventive Medicine.

[ref-9] Catucci A, Scognamiglio U, Rossi L (2021). Lifestyle changes related to eating habits, physical activity, and weight status during COVID-19 quarantine in italy and some European countries. Frontiers in Nutrition.

[ref-10] Centers for Disease Control and Prevention (CDC) (2022). Physical activity for a healthy weight. https://www.cdc.gov/healthyweight/physical_activity/.

[ref-11] Censi L, Ruggeri S, Galfo M, Buonocore P, Roccaldo R (2022). Eating behaviour, physical activity and lifestyle of italian children during lockdown for COVID-19. International Journal of Food Sciences and Nutrition.

[ref-12] Cheval B, Sivaramakrishnan H, Maltagliati S, Fessler L, Forestier C, Sarrazin P, Orsholits D, Chalabaev A, Sander D, Ntoumanis N, Boisgontier MP (2021). Relationships between changes in self-reported physical activity, sedentary behaviour and health during the coronavirus (COVID-19) pandemic in france and switzerland. Journal of Sports Sciences.

[ref-13] Dauchet L, Amouyel P, Hercberg S, Dallongeville J (2006). Fruit and vegetable consumption and risk of coronary heart disease: a meta-analysis of cohort studies. The Journal of Nutrition.

[ref-14] De Veirman M, Cauberghe V, Hudders L (2017). Marketing through instagram influencers: the impact of number of followers and product divergence on brand attitude. International Journal of Advertising.

[ref-15] de Wit L, van Straten A, Lamers F, Cuijpers P, Penninx B (2011). Are sedentary television watching and computer use behaviors associated with anxiety and depressive disorders?. Psychiatry Research.

[ref-16] Delfino LD, dos Santos Silva DA, Tebar WR, Zanuto EF, Codogno JS, Fernandes RA, Christofaro DG (2018). Screen time by different devices in adolescents: association with physical inactivity domains and eating habits. The Journal of Sports Medicine and Physical Fitness.

[ref-17] Deschasaux-Tanguy M, Druesne-Pecollo N, Esseddik Y, de Edelenyi FS, Allès B, Andreeva VA, Baudry J, Charreire H, Deschamps V, Egnell M, Fezeu LK, Galan P, Julia C, Kesse-Guyot E, Latino-Martel P, Oppert J-M, Péneau S, Verdot C, Hercberg S, Touvier M (2021). Diet and physical activity during the coronavirus disease 2019 (COVID-19) lockdown (March–May 2020): results from the french nutrinet-santé cohort study. The American Journal of Clinical Nutrition.

[ref-18] Di Renzo L, Gualtieri P, Pivari F, Soldati L, Attinà A, Cinelli G, Leggeri C, Caparello G, Barrea L, Scerbo F, Esposito E, De Lorenzo A (2020). Eating habits and lifestyle changes during COVID-19 lockdown: an italian survey. Journal of Translational Medicine.

[ref-19] El Ansari W, Adetunji H, Oskrochi R (2014). Food and mental health: relationship between food and perceived stress and depressive symptoms among university students in the United Kingdom. Central European Journal of Public Health.

[ref-20] Frieiro P, González-Rodríguez R, Domínguez-Alonso J (2022). Self-esteem and socialisation in social networks as determinants in adolescents’ eating disorders. Health & Social Care in the Community.

[ref-21] Galafate C (2020). Expansión informe: los efectos del coronavirus en la cesta de la compra de los españoles. https://www.expansion.com/fueradeserie/gastro/2020/04/20/5e9426e5e5fdea656e8b45d7.html.

[ref-22] Gobierno de Cantabria (2021). Resolución por la que se aprueba la decimotercera modificación de la resolución de 18 de junio de 2020, por la que se establecen las medidas sanitarias aplicables en la comunidad autónoma de cantabria durante el período de nueva normalidad, y se adoptan M.

[ref-23] Gobierno de España (2020a). Real decreto 463/2020, de 14 de marzo, por el que se declara el estado de alarma para la gestión de la situación de crisis sanitaria ocasionada por el COVID-19. https://www.boe.es/eli/es/rd/2020/03/14/463.

[ref-24] Gobierno de España (2020b). Plan para la transición hacia una nueva normalidad.

[ref-25] Griffiths LJ, Dowda M, Dezateux C, Pate R (2010). Associations between Sport and Screen-Entertainment with mental health problems in 5-year-old children. International Journal of Behavioral Nutrition and Physical Activity.

[ref-26] Gutierrez J, Willoughby DS (2010). Slimming slumber? How sleep deprivation manipulates appetite and weight. Nutrition Today.

[ref-27] Hale L, Guan S (2015). Screen time and sleep among school-aged children and adolescents: a systematic literature review. Sleep Medicine Reviews.

[ref-29] Hinojo-Lucena F-J, Aznar-Díaz I, Cáceres-Reche M-P, Trujillo-Torres J-M, Romero-Rodríguez J-M (2019). Problematic internet use as a predictor of eating disorders in students: a systematic review and meta-analysis study. Nutrients.

[ref-30] Instituto de Alimentación (INDA) del Ministerio de Desarrollo Social (2020). Cambios en la alimentación de la población de sectores de ingresos medios y altos en el marco de la crisis generada por el coronavirus (COVID-19) en uruguay.

[ref-31] Jacob L, Smith L, Armstrong NC, Yakkundi A, Barnett Y, Butler L, McDermott DT, Koyanagi A, Shin JIl, Meyer J, Firth J, Remes O, López-Sánchez GF, Tully MA (2021). Alcohol use and mental health during COVID-19 lockdown: a cross-sectional study in a sample of UK adults. Drug and Alcohol Dependence.

[ref-32] Joseph RP, Pituch KA, Guest MA, Maxfield M, Peckham A, Coon DW, Kim W, Langer SL (2021). Physical activity among predominantly white middle-aged and older US adults during the SARS-CoV-2 pandemic: results from a national longitudinal survey. Frontiers in Public Health.

[ref-33] Khouja JN, Munafò MR, Tilling K, Wiles NJ, Joinson C, Etchells PJ, John A, Hayes FM, Gage SH, Cornish RP (2019). Is screen time associated with anxiety or depression in young people? Results from a UK birth cohort. BMC Public Health.

[ref-34] King NA, Horner K, Hills AP, Byrne NM, Wood RE, Bryant E, Caudwell P, Finlayson G, Gibbons C, Hopkins M, Martins C, Blundell JE (2012). Exercise, appetite and weight management: understanding the compensatory responses in eating behaviour and how they contribute to variability in exercise-induced weight loss. British Journal of Sports Medicine.

[ref-35] Knell G, Robertson MC, Dooley EE, Burford K, Mendez KS (2020). Health behavior changes during COVID-19 pandemic and subsequent “stay-at-home” orders. International Journal of Environmental Research and Public Health.

[ref-36] Knutson KL (2012). Does inadequate sleep play a role in vulnerability to obesity?. American Journal of Human Biology.

[ref-37] LimeSurvey (2022). LimeSurvey manual. https://manual.limesurvey.org/.

[ref-38] Mattioli AV, Sciomer S, Cocchi C, Maffei S, Gallina S (2020). Quarantine during COVID-19 outbreak: changes in diet and physical activity increase the risk of cardiovascular disease. Nutrition, Metabolism and Cardiovascular Diseases.

[ref-39] McCarthy H, Potts HWW, Fisher A (2021). Physical activity behavior before, during, and after COVID-19 restrictions: longitudinal smartphone-tracking study of adults in the United Kingdom. Journal of Medical Internet Research.

[ref-40] Ministerio de Agricultura, P. y A (2020). Nota de prensa 15 de mayo de 2020. aumenta la presencia de carnes, hortalizas y lácteos en la cesta de la compra de los españoles durante el mes de marzo.

[ref-41] Moitra P, Madan J (2022). Impact of screen time during COVID-19 on eating habits, physical activity, sleep, and depression symptoms: a cross-sectional study in indian adolescents. PLOS ONE.

[ref-42] Moynihan AB, Tilburg WAP, Igou ER, Wisman A, Donnelly AE, Mulcaire JB (2015). Eaten up by boredom: consuming food to escape awareness of the bored self. Frontiers in Psychology.

[ref-43] Muscogiuri G, Barrea L, Savastano S, Colao A (2020). Nutritional recommendations for CoVID-19 quarantine. European Journal of Clinical Nutrition.

[ref-44] Organización Panamericana de la Salud (2020). La OMS caracteriza a COVID-19 como una pandemia. https://www.paho.org/es/noticias/11-3-2020-oms-caracteriza-covid-19-como-pandemia.

[ref-45] Park E-J, Hwang SS-H, Lee M-S, Bhang S-Y (2022). Food addiction and emotional eating behaviors co-occurring with problematic smartphone use in adolescents?. International Journal of Environmental Research and Public Health.

[ref-46] Pérez-Rodrigo C, Gianzo- Citores M, Hervás Bárbara G, Ruiz-Litag F, Casis-Sáenz L, Aranceta-Bartrina J, Grupo, Val VA, López-Sobaler AM, de Victoria EM, Ortega RM, Partearroyo T, Quiles J (2020). Cambios en los hábitos alimentarios durante el periodo de confinamiento por la pandemia COVID-19 en españa. Revista Espanola de Nutricion Comunitaria.

[ref-47] Ramón-Arbués E, Martínez Abadía B, Granada López JM, Echániz Serrano E, Pellicer García B, Juárez Vela R, Guerrero Portillo S, Saéz Guinoa M (2019). Eating behavior and relationships with stress, anxiety, depression and insomnia in university students. Nutrición Hospitalaria.

[ref-48] Redman LM, Heilbronn LK, Martin CK, de Jonge L, Williamson DA, Delany JP, Ravussin E (2009). Metabolic and behavioral compensations in response to caloric restriction: implications for the maintenance of weight loss. PLOS ONE.

[ref-49] Revista ConsUCE (2020). Hábitos de consumo durante el COVID-19. https://uniodeconsumidors.org/es/revista-consuce-47-habitos-de-consumo-durante-el-covid19/.

[ref-50] Robinson E, Boyland E, Chisholm A, Harrold J, Maloney NG, Marty L, Mead BR, Noonan R, Hardman CA (2021). Obesity, eating behavior and physical activity during COVID-19 lockdown: a study of UK adults. Appetite.

[ref-51] Rodríguez M, Crespo I, Olmedillas H (2020). Ejercitarse en tiempos de la COVID-19: ¿qué recomiendan hacer los expertos entre cuatro paredes?. Revista Española de Cardiología.

[ref-52] Severi C, Medina M (2020). Cambios en los hábitos alimentarios y actividad física durante el aislamiento físico durante el COVID-19. Anales De La Facultad De Medicina.

[ref-53] Tan M, He FJ, MacGregor GA (2020). Obesity and covid-19: the role of the food industry. BMJ.

[ref-54] Tayhan Kartal F, Yabancı Ayhan N (2021). Relationship between eating disorders and internet and smartphone addiction in college students. Eating and Weight Disorders—Studies on Anorexia, Bulimia and Obesity.

[ref-55] Teychenne M, Costigan SA, Parker K (2015). The association between sedentary behaviour and risk of anxiety: a systematic review. BMC Public Health.

[ref-56] Villaseñor Lopez K, Jimenez Garduño AM, Ortega Regules AE, Islas Romero LM, Gonzalez Martinez OA, Silva Pereira TS (2021). Cambios en el estilo de vida y nutrición durante el confinamiento por SARS-CoV-2 (COVID-19) en méxico: un estudio observacional. Revista Española de Nutrición Humana y Dietética.

[ref-57] Wang X, Ouyang Y, Liu J, Zhu M, Zhao G, Bao W, Hu FB (2014). Fruit and vegetable consumption and mortality from all causes, cardiovascular disease, and cancer: systematic review and dose-response meta-analysis of prospective cohort studies. BMJ.

[ref-58] Wang C, Pan R, Wan X, Tan Y, Xu L, Ho CS, Ho RC (2020). Immediate psychological responses and associated factors during the initial stage of the 2019 coronavirus disease (COVID-19) epidemic among the general population in China. International Journal of Environmental Research and Public Health.

[ref-59] Wang Y, Zhu L-Y, Ma Y-F, Bo H-X, Deng H-B, Cao J, Wang Y, Wang X-J, Xu Y, Lu Q-D, Wang H, Wu X-J (2020). Association of insomnia disorder with sociodemographic factors and poor mental health in COVID-19 inpatients in China. Sleep Medicine.

[ref-60] Warburton DER, Bredin SSD (2017). Health benefits of physical activity. Current Opinion in Cardiology.

[ref-61] World Health Organization (WHO) (2020). Healthy diet. https://www.who.int/news-room/fact-sheets/detail/healthy-diet.

[ref-62] World Health Organization (WHO) (2010). Global recommendations on physical activity for health. https://www.who.int/publications/i/item/9789241599979.

[ref-63] Yu E, Malik VS, Hu FB (2018). Cardiovascular disease prevention by diet modification. Journal of the American College of Cardiology.

[ref-64] Zhou S-J, Wang L-L, Yang R, Yang X-J, Zhang L-G, Guo Z-C, Chen J-C, Wang J-Q, Chen J-X (2020). Sleep problems among chinese adolescents and young adults during the coronavirus-2019 pandemic. Sleep Medicine.

