# Supplementary material for: Change in eating habits during the Spanish COVID-19 pandemic lockdown: evidence for a sample of university community
Source: PeerJ. 2023 Jan 25;11:e14244. doi: 10.7717/peerj.14244 (PMC9884042; doi:10.7717/peerj.14244)
Supplement: Supplemental Information 2 [file peerj-11-14244-s002.docx]

***Título:***

***Cambios en estilos de vida y distancia social durante la pandemia de COVID-19***

El objetivo del estudio es analizar el efecto de la COVID-19 sobre varios aspectos de hábitos/estilos de vida en los diferentes colectivos de la Universidad de Cantabria. Para obtener la información necesaria, se realizará una breve encuesta al alumnado de la Universidad de Cantabria, así como al personal de la misma (PDI y PAS) en la que se abarcarán diversas preguntas que describan a la población objeto de estudio.

A continuación, se muestran las preguntas que se incluirán en el cuestionario online. Estas aparecerán después de que el encuestado haya leído la hoja informativa y aceptado el modelo de consentimiento.

**PARTE I: DATOS PERSONALES**

1. **Género:**
   - Hombre.
   - Mujer.
   - Otro.
2. **Edad (en años): ________**
3. **Grupo de población al que pertenece.**
   - Estudiante.
     - Grado.
     - Postgrado.
     - Senior.
   - Personal que trabaja en UC (Personal de Administración y Servicios y Personal Docente e Investigador).
4. **Código postal de su residencia habitual.**
   - ________________
5. **Estado civil:**
   - Soltero/a.
   - Casado/a o pareja de hecho.
   - Viudo/a.
   - Separado/a o Divorciado/a.
6. **¿Tiene a su cargo el cuidado de ascendientes o descendientes?**
   - Sí.
   - No.
7. **¿Cuántas personas residen en el hogar? Inclúyase usted mismo.**
   - 1.
   - 2.
   - 3.
   - 4.
   - 5
   - Más de 5.

**PARTE II: DISTANCIA SOCIAL**

1. **Para prevenir el contagio, ¿cuál de estos comportamientos cotidianos está adoptando? Indique las que aplican en su caso:**
   - Me lavo las manos con mucha frecuencia.
   - Siempre utilizo mascarilla.
   - Trato de mantener una distancia de seguridad que me separe de la gente.
   - Voy a los supermercados lo menos posible.
   - Ya no quedo con no convivientes.
   - No salgo de casa salvo en caso de emergencia.
   - Ninguno de los anteriores.
   - Otros. Indique cuáles.
   - Prefiero no contestar.
2. **De lunes a viernes, señale los motivos por los que sale usted de casa:**
   - Trabajo.
   - Compras en supermercados.
   - Compras en general.
   - Provisión de cuidados a personas dependientes.
   - Ocio en lugares al aire libre.
   - Ocio en lugares cerrados.
   - No salgo de casa.
3. **Los fines de semana, señale los motivos por los que sale usted de casa:**
   - Trabajo.
   - Compras en supermercados.
   - Compras en general.
   - Provisión de cuidados a personas dependientes.
   - Ocio en lugares al aire libre.
   - Ocio en lugares cerrados.
   - No salgo de casa.
4. **Antes de la COVID19, ¿Cuántas veces a la semana iba a una terraza, bar, restaurante o similar?**
   - 0
   - 1
   - 2
   - 3
   - Más de 3.
5. **Actualmente con la situación de la COVID19, ¿Cuántas veces a la semana va a una terraza, bar, restaurante o similar?**
   - 0
   - 1
   - 2
   - 3
   - Más de 3.
6. **Antes de la COVID19, ¿cuántas veces a la semana quedaba con amigos/familiares en casas, garajes, locales etc.?**
   - 0
   - 1
   - 2
   - 3
   - Más de 3.
7. **Actualmente con la situación de la COVID19, ¿Cuántas veces a la semana queda con amigos/familiares en casas, garajes, locales etc.?**
   - 0
   - 1
   - 2
   - 3
   - Más de 3.
8. **¿En qué medida cree que las personas de su entorno cumplen con la normativa y medidas establecidas para la contención del virus? Valore de 0 a 10 (cero, no cumplen; diez, pleno cumplimiento).**
9. **¿Cómo de preocupado/a estaría si usted o algún familiar enfermara de coronavirus?**
   - Muy preocupado.
   - Algo preocupado.
   - No demasiado preocupado.
   - No está preocupado en absoluto.
10. **¿Su forma de actuar se vería afectada si las multas y/o sanciones fueran disuasorias?**
    - Si.
    - No.
11. **En lo referente a la COVID-19, ¿Cuánto confía en lo que dicen los profesionales sanitarios y expertos en salud? Valore de 0 a 10 (cero, nada de confianza; diez, plena confianza).**
12. **En lo referente a la COVID-19, ¿Cuánto confía en lo que dicen los medios de comunicación? Valore de 0 a 10 (cero, nada de confianza; diez, plena confianza).**

**PARTE III: ESTADO DE SALUD**

1. **¿Cómo considera su salud general?**
   - Muy buena.
   - Buena.
   - Regular.
   - Mala.
   - Muy mala.
2. **Comparando la situación previa al confinamiento y la actual, ¿ha variado su salud general?**
   - Sí, ha mejorado.
   - Sí, ha empeorado.
   - No, se ha mantenido.

**PARTE IV: HÁBITO ALIMENTARIO**

1. **¿Cómo considera sus hábitos alimenticios?**
   - Muy buenos.
   - Buenos.
   - Regulares.
   - Malos.
   - Muy malos.
2. **Comparando la situación previa al confinamiento y la actual, ¿han variado sus hábitos alimenticios?**
   - Sí, han mejorado.
   - Sí, han empeorado.
   - No, se han mantenido.
3. **Comparando la situación previa al confinamiento y la actual, ¿ha variado su peso?**
   - Sí, ha subido.
   - Sí, ha bajado.
   - No, se ha mantenido.
4. **¿Sigue alguna dieta específica?**
   - Sí.
   - No.
5. **¿Con qué frecuencia semanal consume los siguientes productos?**

|  | **Diariamente** | **5 o 6 veces por semana** | **3 o 4 veces por semana** | **1 o 2 veces por semana** | **Una o varias veces al mes** | **Nunca** |
| --- | --- | --- | --- | --- | --- | --- |
| **Bebidas azucaradas** |  |  |  |  |  |  |
| **Dulces** |  |  |  |  |  |  |
| **Alcohol** |  |  |  |  |  |  |
| **Frutas y verduras** |  |  |  |  |  |  |

**PARTE V: “OTROS”**

1. **¿Le gusta asumir riesgos? Valore de 0 a 10 (cero, no me gustan los riesgos; diez, “no tengo miedo a nada”).**
2. **Comparando la situación previa a la pandemia de COVID19 y la actual, ¿ha variado su tiempo dedicado al estudio/trabajo?**
   - Sí, ha subido ese número de horas que dedico a esas tareas.
   - Sí, ha bajado ese número de horas que dedico a esas tareas.
   - No, se ha mantenido.
3. **Aproximadamente, ¿a qué intervalo de ingresos mensuales corresponde el de su unidad familiar?**
   - Menos de 748,7€ mensuales.
   - De 748,7€ a 1.046,9€ mensuales.
   - De 1.046,9€ a 1.260,9€ mensuales.
   - De 1.260,9€ a 1.453,6€ mensuales.
   - De 1.453,6€ a 1.642,3€ mensuales.
   - De 1.642,3€ a 1.871,3€ mensuales.
   - De 1.871,3€ a 2.187,2€ mensuales.
   - De 2.187,2€ a 2.674,7€ mensuales.
   - De 2.674,7€ a 3.441,9€ mensuales.
   - 3.441,9€ o más.
   - No sabe/no contesta.
4. **Número de horas semanales que dedica/dedicaba a la práctica de deporte.**

|  | **Antes del confinamiento** | **Después del confinamiento** |
| --- | --- | --- |
| No realizo deporte. |  |  |
| Menos de 4 horas semanales. |  |  |
| Entre 4 y 6 horas semanales. |  |  |
| Entre 6 y 8 horas semanales. |  |  |
| Más de 8 horas semanales. |  |  |

1. **Lugar donde practica/practicaba deporte con mayor frecuencia**

|  | **Antes del confinamiento** | **Después del confinamiento** |
| --- | --- | --- |
| No realizo deporte. |  |  |
| En un gimnasio, centro deportivo y/o similar |  |  |
| En casa. |  |  |
| Al aire libre |  |  |

1. **¿Han cambiado sus hábitos tabáquicos con la pandemia de la COVID19?**
   - Sí, he empezado a fumar.
   - Sí, fumo más.
   - Sí, fumo menos.
   - No, fumo lo mismo.
   - No fumo.
2. **¿Han cambiado sus hábitos respecto al uso del teléfono móvil con la pandemia de la COVID19?**
   - Sí, lo usó más tiempo.
   - Sí, lo usó menos tiempo.
   - No, lo usó el mismo tiempo.
   - Prácticamente no lo usó.
3. **Durante el día ¿Cuántas horas puede estar sin seguir pendiente del teléfono móvil (nomofobia: intervalo de tiempo sin mirar el teléfono móvil)?**
   - Más de 5 horas sin mirarlo.
   - 5 horas sin mirarlo.
   - 4 horas sin mirarlo.
   - 3 horas sin mirarlo.
   - 2 horas sin mirarlo.
   - 1 hora sin mirarlo.
   - Menos de 1 hora sin mirarlo.
