# Supplementary material for: Change in eating habits during the Spanish COVID-19 pandemic lockdown: evidence for a sample of university community
Source: PeerJ. 2023 Jan 25;11:e14244. doi: 10.7717/peerj.14244 (PMC9884042; doi:10.7717/peerj.14244)
Supplement: Supplemental Information 3 [file peerj-11-14244-s003.pdf]

Changes in lifestyles and social distance during the COVID-19 pandemic

The aim of the study is to analyse the effect of COVID-19 on various aspects of habits/lifestyles in different groups at the University of Cantabria. In order to obtain the necessary information, a brief survey will be carried out among the students of the University of Cantabria, as well as its staff (Administration and Services Staff and Teaching and Research Staff) in which several questions describing the population under study will be covered.

The questions to be included in the online questionnaire are shown below. These will appear after the respondent has read the information sheet and accepted the consent form.

SECTION I: PERSONAL DATA

1. **Gender:**
   - ☐ Man.
   - ☐ Woman.
   - ☐ ____

2. **Age: ________**

3. **Population group to which it belongs.**
   - ☐ Student.
     - ☐ Degree.
     - ☐ Postgraduate.
     - ☐ Senior.
   - ☐ Staff working at the University of Cantabria (Administration and Services Staff and Teaching and Research Staff).

4. **Postal code of your usual residence.**
   - ☐ ________________

5. **Marital status:**
   - ☐ Single.
   - ☐ Married or with a partnership.
   - ☐ Widowed.
   - ☐ Separated or divorced.

6. **Are you responsible for the care of relatives in the ascending or descending line?**
   - ☐ Yes.

- ☐ No.

7. How many persons reside in the household? Include yourself.

- ☐ 1.
- ☐ 2.
- ☐ 3.
- ☐ 4.
- ☐ 5
- ☐ More than 5.

SECTION II: SOCIAL DISTANCING

8. To prevent infection, which of these everyday behaviours are you adopting? Indicate the ones that apply to you:

- ☐ I wash my hands very often.
- ☐ I always wear a mask.
- ☐ I try to keep a safe distance away from people.
- ☐ I go to supermarkets as little as possible.
- ☐ I no longer meet non-cohabitants.
- ☐ do not leave the house except in case of emergency.
- ☐ None of the above.
- ☐ Other. Please indicate which ones.
- ☐ I prefer not to answer.

9. From Monday to Friday, please indicate the reasons why you go out of the house:

- ☐ Work.
- ☐ Shopping in supermarkets.
- ☐ General shopping.
- ☐ Provision of care for dependants.
- ☐ Outdoor leisure.
- ☐ Leisure indoors.
- ☐ I do not go out of the house.

10. On weekends, please indicate the reasons why you go out of your home:

- ☐ Work.
- ☐ Shopping in supermarkets.
- ☐ General shopping.
- ☐ Provision of care for dependants.
- ☐ Outdoor leisure.

- ☐ Leisure indoors.
- ☐ I do not go out of the house.

11. Before COVID19, how many times a week did you go to a terrace, bar, restaurant or similar?

- ☐ 0
- ☐ 1
- ☐ 2
- ☐ 3
- ☐ More than 3.

12. Currently with the COVID19 situation, how many times a week do you go to a terrace, bar, restaurant or similar?

- ☐ 0
- ☐ 1
- ☐ 2
- ☐ 3
- ☐ More than 3.

13. Before COVID19, how many times a week did you meet friends/relatives in houses, garages, premises etc.?

- ☐ 0
- ☐ 1
- ☐ 2
- ☐ 3
- ☐ More than 3.

14. Currently with the COVID19 situation, how many times a week do you stay with friends/relatives in houses, garages, premises etc.?

- ☐ 0
- ☐ 1
- ☐ 2
- ☐ 3
- ☐ More than 3.

15. To what extent do you think that the people around you comply with the regulations and measures established for the containment of the virus? Please rate from 0 to 10 (zero, non-compliant; ten, fully compliant).

- ☐

16. How worried would you be if you or a family member became ill with coronavirus?

- ☐ Very concerned.

☐ Somewhat concerned.
☐ Not too worried.
☐ Not worried at all.

17. **Would your behaviour be affected if fines and/or sanctions were dissuasive?**
    ☐ Yes.
    ☐ No.

18. **How much confidence do you have in what health professionals and health experts say about COVID-19? Please rate from 0 to 10 (zero, no confidence; ten, full confidence).**
    ☐

19. **How much confidence do you have in what the media says about COVID-19? Please rate from 0 to 10 (zero, no confidence; ten, full confidence).**
    ☐

SECTION III: HEALTH STATUS

20. **¿ How do you consider your general health?**
    □ Very good.
    □ Good.
    □ Fair.
    □ Bad.
    □ Very bad.

21. **Comparing your pre-confinement situation and your current situation, has your general health changed?**
    □ Yes, it has improved.
    □ Yes, it has worsened.
    □ No, it has remained the same.

SECTION IV: EATING HABITS

22. **¿ How you view your eating habits?**
    ☐ Very good.
    ☐ Good.
    ☐ Fair.
    ☐ Bad.
    ☐ Very bad.

23. **Comparing the situation before lockdown and now, have your eating habits changed?**

□ Yes, it has improved.
□ Yes, it has worsened.
□ No, it has remained the same.

24. **Comparing the pre-lockdown situation and the current situation, has your weight changed?**
    ☐ Yes, it has gone up.
    ☐ Yes, it has gone down.
    ☐ No, it has stayed the same.

25. **Do you follow a specific diet?**
    ☐ Yes.
    ☐ No.

26. **How often per week do you consume the following products?**

	**Daily**	**5 or 6 times per week**	**3 or 4 times per week**	**1 or 2 times per week**	**Once or several times a month**	**Never**
**Sugary drinks**						
**Sweets**						
**Alcohol**						
**Fruits and vegetables**						

SECTION V: "OTHER"

27. **Do you like to take risks? Rate from 0 to 10 (zero, I don't like risks; ten, "I'm not afraid of anything").**

28. **Comparing the situation before the COVID19 pandemic and the current situation, has your study/work time changed?**
    ☐ Yes, the number of hours I spend on these tasks has increased.
    ☐ Yes, the number of hours I spend on these tasks has decreased.
    ☐ No, it has remained the same.

29. **Approximately what is the monthly income range of your household?**
    ☐ Less than €748.7 per month.
    ☐ From €748.7 to €1,046.9 per month.
    ☐ From €1,046.9 to €1,260.9 per month.

- ☐ From €1,260.9 to €1,453.6 per month.
- ☐ From €1,453.6 to €1,642.3 per month.
- ☐ From €1,642.3 to €1,871.3 per month.
- ☐ From €1,871.3 to €2,187.2 per month.
- ☐ From €2,187.2 to €2,674.7 per month.
- ☐ From €2,674.7 to €3,441.9 per month.
- ☐ 3,441.9€ or more.
- ☐ Don't know/no answer.

30. Number of hours per week spent/dedicated to sports practice.

	**Before lockdown**	**After lockdown**
**I do not do sport.**		
**Less than 4 hours per week.**		
**Between 4 and 6 hours per week.**		
**Between 6 and 8 hours per week.**		
**More than 8 hours per week.**		

31. Place where you practice/used to practice sport most frequently.

	**Before lockdown**	**After lockdown**
**I do not do sport.**		
**In a gym, sports centre and/or similar**		
**At home.**		
**Outdoors**		

32. Have your smoking habits changed with the COVID19 pandemic?

- ☐ Yes, I have started smoking.
- ☐ Yes, I smoke more.
- ☐ Yes, I smoke less.
- ☐ No, I smoke the same.
- ☐ I do not smoke.

33. Have your mobile phone habits changed with the COVID19 pandemic?

- ☐ Yes, used it longer.
- ☐ Yes, used it less time.

☐ No, he used it the same amount of time.
☐ Practically did not use it at all.

34. During the day, how many hours can you spend without looking at your mobile phone (nomophobia: time interval without looking at your mobile phone)?

☐ More than 5 hours without looking at it.
☐ 5 hours without looking at it.
☐ 4 hours without looking at it.
☐ 3 hours without looking at it.
☐ 2 hours without looking at it.
☐ 1 hour without looking at it.
☐ Less than 1 hour without looking at it.
